# Relationship between leukocyte counts and large vessel occlusion in acute ischemic stroke

**DOI:** 10.1186/s12883-020-02017-3

**Published:** 2020-12-04

**Authors:** Gabor Tarkanyi, Zsofia Nozomi Karadi, Zsofia Szabo, Istvan Szegedi, Laszlo Csiba, Laszlo Szapary

**Affiliations:** 1 Department of Neurology, University of Pécs, Ifjúság u. 13, Pécs, 7624 Hungary; 2 Department of Neurology, University of Debrecen, Debrecen, Hungary

**Keywords:** Ischemic stroke, Large vessel occlusion, Leukocytes, Neutrophils, Neuroinflammation

## Abstract

**Background:**

Neuroinflammation plays an important role in the pathogenesis of acute ischemic stroke (AIS) and peripheral leukocyte counts have proved to be independent predictors of stroke severity and outcomes. Clinical significance of large vessel occlusion (LVO) in AIS is increasing, as these patients are potential candidates for endovascular thrombectomy and likely to have worse outcomes if not treated urgently. The aim of our study was to assess the relationship between on admission leukocyte counts and the presence of LVO in the early phase of AIS.

**Methods:**

We have conducted a cross-sectional, observational study based on a registry of consecutive AIS patients admitted up to 4.5 h after stroke onset. Blood samples were taken at admission and leukocyte counts were measured immediately. The presence of LVO was verified based on the computed tomography angiography scan on admission.

**Results:**

Total white blood cell (WBC) and neutrophil counts were significantly higher in patients with LVO than those without LVO (*P* < 0.001 respectively). After adjustment for potential confounders total WBC counts (adjusted OR: 1.405 per 1 × 10^9^/L increase, 95% CI: 1.209 to 1.632) and neutrophil counts (adjusted OR: 1.344 per 1 × 10^9^/L increase, 95% CI: 1.155 to 1.564) were found to have the strongest associations with the presence of LVO. Total WBC and neutrophil counts had moderate ability to discriminate an LVO in AIS (AUC: 0.667 and 0.655 respectively). No differences were recorded in leukocyte counts according to the size of the occluded vessel and the status of collateral circulation in the anterior vascular territory. However, total WBC and neutrophil counts tended to be higher in patients with LVO in the posterior circulation (*p* = 0.005 and 0.010 respectively).

**Conclusion:**

Higher admission total WBC and neutrophil counts are strongly associated with the presence of LVO and has moderate ability to discriminate an LVO in AIS. Detailed evaluation of stroke-evoked inflammatory mechanisms and changes according to the presence of LVO demands further investigation.

**Supplementary Information:**

The online version contains supplementary material available at 10.1186/s12883-020-02017-3.

## Background

Secondary neuroinflammation plays an important role in the pathogenesis of acute ischemic stroke (AIS). Ischemic brain damage elicits systematic inflammatory response and cause a time-dependent activation of peripheral immune cells [1]. Leukocyte counts and ratios (such as neutrophil-to-lymphocyte ratio) in peripheral blood proved to have good prognostic value to predict outcomes and post-stroke complications [2, 3]. Higher leukocyte counts, especially neutrophil elevation is also associated with increasing severity and larger infarct volumes in AIS [4, 5].

Approximately 20 to 40% of AIS cases are caused by large vessel occlusion (LVO), early detection of which is crucial because these patients are potential candidates for endovascular thrombectomy (EVT) and have worse outcomes if not treated urgently [6, 7]. Large vessel occlusion tends to cause more severe strokes and place large cerebral territories at ischemic risk [8]. Therefore, the magnitude of peripheral inflammatory response may be related to the presence of LVO, however previous studies did not investigate this context. The aim of our study was to examine the relationship between on admission total and differential leukocyte counts and the presence of LVO in the early phase of AIS.

## Methods

### Study population

We have conducted a cross-sectional, observational study based on a prospectively collected registry of consecutive AIS patients admitted up to 4.5 h after symptom onset to the comprehensive stroke centres (CSC) of two university hospitals between October 2017 and October 2019. Blood samples were collected on admission. Total and differential leukocyte counts were measured immediately with an automated hemocytometer (Sysmex XN-1000; Sysmex, Kobe, Japan). We have recorded demographic data, vascular risk factors, baseline clinical variables, baseline laboratory values, medications at stroke onset and times from onset to sample collection for each patient. On admission stroke severity was assessed using the National Institutes of Health Stroke Scale (NIHSS).

Our outcome of interest was the presence of LVO on the admission computed tomography angiography (CTA) scan. According to *Rennert* et al. [9] unilateral, acute occlusion of the internal carotid artery (ICA), M1, M2 and M3 segments of the middle cerebral artery (MCA), A1 and A2 segments of the anterior cerebral artery (ACA), vertebral artery (VA), basilar artery (BA), P1 and P2 segments of the posterior cerebral artery (PCA) and tandem occlusions were considered. Collateral circulation in the anterior vascular territory was evaluated using the multiphase CTA (mCTA) collateral score. Patients were dichotomized into two groups according to good (mCTA 4–5 points) and poor (mCTA 0–3 points) collateral circulation [10]. Evaluation of CTA scan and mCTA collateral score was done by trained neuroradiologist as a standard of care who were blinded to clinical data. Data on early post-stroke infections (PSI) were recorded considering any type of infection occurred within 72 h from stroke onset and were at least Grade 2 in severity according to Common Terminology Criteria for Adverse Events [11].

Patients without CTA assessment or whose laboratory results were missing due to sampling or measurement errors were excluded. We have also excluded patients who had infection or surgery within 2 weeks prior to the stroke, those who had relevant neurological events (transient ischemic attack [TIA] before or seizures after stroke onset), those who take immunomodulatory medications and those with haematological malignancies, as these conditions could influence peripheral leukocyte counts.

### Statistical analysis

Data analysis was performed using SPSS (version 26.0, IBM, New York). Continuous variables were presented as mean and standard deviation (SD) or as median and interquartile range (IQR) where appropriate. Categorical variables were presented as counts and percentages. In the univariate analysis the comparison of continuous variables was performed using *t* test or *Mann-Whitney U* test. Normality was assessed using the *Shapiro-Wilk* test and visually, based on Q-Q plots and histograms. Categorical data were compared using the *Pearson X*^*2*^ test or the *Fischer* exact test when expected value in any cell was below 5. Univariable and multivariable binary logistic regression analysis was performed to assess the associations between leukocyte counts and the presence of LVO, variables with *P* value ≤0.1 in the univariable analysis were included in the multivariable model. Total white blood cell (WBC) count, each leukocyte subtype counts and neutrophil-to-lymphocyte ratio (NLR) were entered in a separate model because of multicollinearity. The ability of leukocyte counts to discriminate the presence of LVO was assessed using the receiver operating characteristic analysis, area under the curve (AUC) was calculated for each variable. Optimal cut-off values were calculated using *Youden J* statistics. Odds ratios (OR) and 95% confidence intervals (CI) were presented where appropriate, *P* < 0.05 was considered as statistical significance.

## Results

**Fig. 1:**
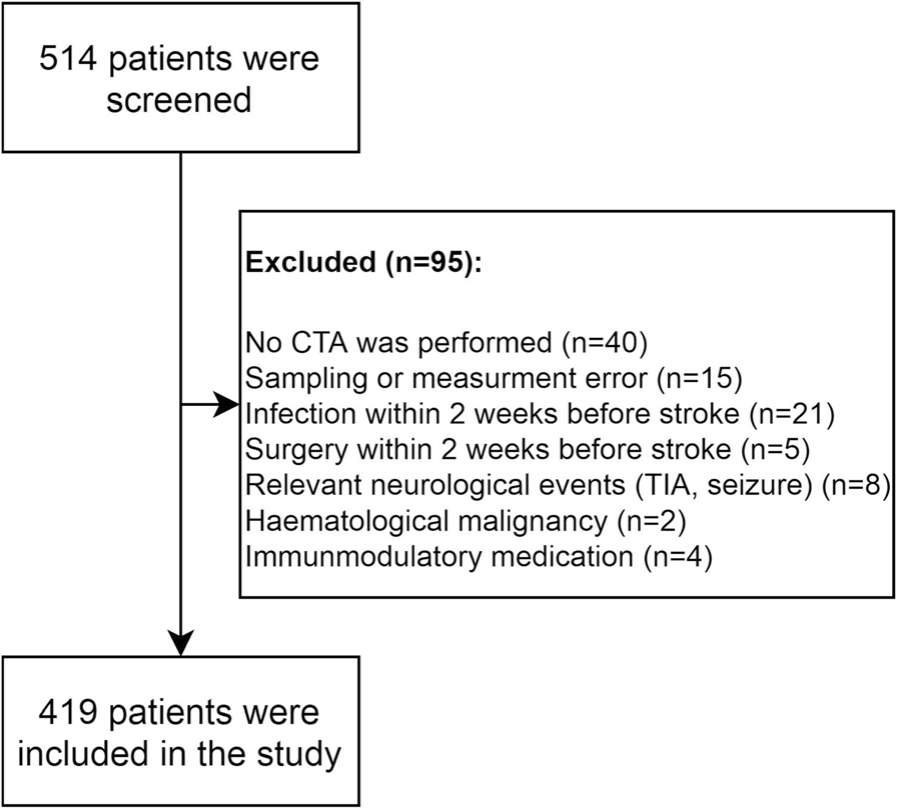
Patient exclusion flowchart

During the study period 514 patients were screened, after exclusions the data of 419 patients were analysed (Fig. 1). The main age of the study cohort was 67.7 ± 12.2 years (43.9% female), 167 patients had LVO (39.9%). Demography and baseline characteristics of the cohort are presented in Table 1. Univariable associations between baseline variables and the presence of LVO are presented in Table S1 of the Supplementary material.

**Table 1:** Demography and clinical characteristics of the cohort according to the presence of LVO

	LVO present (*N* = 167)	LVO absent (*N* = 252)	*P* value
**Demographic characteristics**			
Age, years, median (IQR)	68 (61–79)	69 (59–77)	0.258
Gender, female, % (n)	52.1 (87)	38.5 (97)	**0.006**
**Elapsed times**			
Onset-to-sample time, min, median (IQR)	83 (55–124)	88 (59–139)	0.313
Sample-to-CTA time, min, median (IQR)	16 (6–25)	12 (5–28)	0.684
**Parameters on admission**			
NIHSS score on admission, median (IQR)	12 (7–17)	6 (4–8)	**< 0.001**
On admission SBP, mmHg, median (IQR)	158 (140–177)	167 (145–180)	**0.004**
On admission DBP, mmHg, median (IQR)	85 (78–96)	90 (80–100)	**0.004**
Body temperature, ^o^C, median (IQR)	36.4 (36.1–36.5)	36.4 (36.2–36.6)	0.069
Blood glucose, mmol/L, median (IQR)	6.89 (5.90–8.10)	6.43 (5.61–8.35)	0.120
INR, ratio, median (IQR)	1.02 (0.95–1.08)	0.99 (0.94–1.04)	**0.003**
**Vascular risk factors**			
Smoking, % (n), 60 missing	39.1 (52)	31.4 (71)	0.139
Hypertension, % (n), 13 missing	81.6 (133)	77.8 (189)	0.352
Diabetes mellitus, % (n), 19 missing	21.4 (34)	30.3 (73)	**0.049**
Hyperlipidaemia, % (n), 36 missing	50.7 (76)	53.6 (125)	0.568
Atrial fibrillation, % (n), 23 missing	32.9 (52)	17.2 (41)	**< 0.001**
Coronary artery disease, % (n), 33 missing	27.7 (43)	23.4 (54)	0.332
Chronic heart failure, % (n), 23 missing	15.0 (24)	7.6 (18)	**0.019**
Previous stroke/TIA, % (n), 22 missing	17.6 (28)	25.2 (60)	0.074
Malignancy, % (n), 31 missing	16.4 (25)	9.3 (22)	0.036
**Therapy at stroke onset**			
Antiplatelet, % (n), 23 missing	40.3 (62)	36.0 (87)	0.388
Anticoagulant, % (n), 28 missing	17.6 (27)	9.7 (23)	**0.021**
Lipid lowering, % (n), 23 missing	27.7 (43)	22.4 (54)	0.228
Antihypertensive, % (n), 24 missing	72.9 (113)	66.7 (160)	0.190
Antidiabetic, % (n), 24 missing	16.4 (25)	24.0 (58)	0.070

Abbreviation: *LVO* large vessel occlusion; *NIHSS* National Institutes of Health Stroke Scale; *SBP* systolic blood pressure; *DBP* diastolic blood pressure; *IQR* interquartile range; *INR* International Normalized Ratio; *TIA* transient ischemic attack

**Fig. 2:**
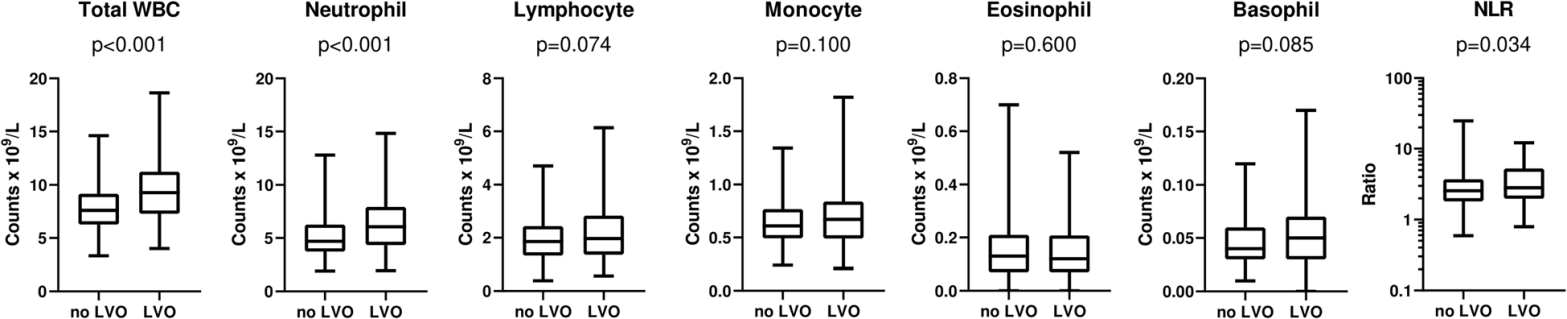
Comparison of admission total white blood cell (WBC) counts, leukocyte subtype counts and neutrophil-to-lymphocyte ratio (NLR) values in acute ischemic stroke according to the presence of large vessel occlusion (LVO). Boxes, 25 to 75% interquartile range; central horizontal bars, median; outer horizontal bars, minimum and maximum values. Statistics: Mann-Whitney *U* test

Higher total WBC counts were recorded in LVO patients than those without LVO (9.27 × 10^9^/L vs. 7.61 × 10^9^/L; *P* < 0.001). Regarding major leukocyte subtypes, median neutrophil counts were significantly higher in the LVO group (6.05 × 10^9^/L vs. 4.69 × 10^9^/L; *P* < 0.001). In contrast, no significant difference was recorded between the groups for the other subtypes (Fig. 2). Neutrophil-to-lymphocyte ratio values was slightly higher in patients with LVO (2.83 versus 2.56; *P* = 0.034). Increasing onset to sample times correlated with higher neutrophil counts (Spearman *r*, 0.175; *P* < 0.001), lower lymphocyte counts (Spearman *r*, − 0.229; *P* < 0.001) and increasing NLR values (Spearman *r*, 0.275; *P* < 0.001).

Univariable binary logistic regression analysis showed associations between on admission total WBC, neutrophil, lymphocyte, monocyte and basophil counts and the presence of LVO. After adjustment for potential confounders independent associations were only found between total WBC, neutrophil, lymphocyte and basophil counts and the presence of LVO (Table 2). There was a trend between increasing NLR values and the presence of LVO in the univariable analysis (OR: 1.079 per 1-point increase, 95% CI: 1.001 to 1.164; *P* = 0.048), but this trend was not present after adjustment for confounders (OR: 1.022 per 1-point increase, 95% CI: 0.924 to 1.131; *P* = 0.672).

**Table 2:** Associations between leukocyte counts and the presence of large vessel occlusion in acute ischemic stroke

	Crude OR (95% CI)	*P* value	Adjusted OR (95% CI)^a^	*P* value
Total WBC (1 × 10^9^/L increase)	1.292 (1.187 to 1.405)	**< 0.001**	1.405 (1.209 to 1.632)	**< 0.001**
Neutrophil (1 × 10^9^/L increase)	1.296 (1.181 to 1.421)	**< 0.001**	1.344 (1.155 to 1.564)	**< 0.001**
Lymphocyte (1 × 10^9^/L increase)	1.321 (1.064 to 1.641)	**0.012**	1.631 (1.106 to 2.407)	**0.014**
Monocyte (0.1 × 10^9^/L increase)	1.112 (1.018 to 1.214)	**0.018**	1.048 (0.903 to 1.217)	0.535
Eosinophil (0.1 × 10^9^/L increase)	0.955 (0.807 to 1.131)	0.596	1.043 (0.799 to 1.363)	0.755
Basophil (0.01 × 10^9^/L increase)	1.106 (1.024 to 1.194)	**0.010**	1.296 (1.119 to 1.501)	**< 0.001**

Abbreviation: *OR* odds ratio; *CI* confidence interval; *WBC* white blood cell; *L* litre

^a^ Adjusted to sex, on admission NIHSS score, systolic blood pressure, diastolic blood pressure, body temperature, INR value, the presence of diabetes mellitus, atrial fibrillation, chronic heart failure, previous stroke/TIA, malignancy in patient history and anticoagulant or antidiabetic therapy at stroke onset

Receiver operating characteristic analyses demonstrated moderate ability of total WBC (AUC: 0.667, 95% CI: 0.613 to 0.721; *P* < 0.001) and neutrophil counts (AUC: 0.655, 95% CI: 0.600 to 0.710; P < 0.001) to discriminate the presence of LVO. Marginally significant ability was detected for NLR values (AUC: 0.563, 95% CI: 0.505 to 0.621; *P* = 0.030), and the abilities of other leukocyte subtypes to discriminate an LVO were not significant (Figure S1 and Table S2 in the Supplementary material).

Out of 167 LVO patients 147 (88.0%) had occlusion in the anterior circulation (ICA, M1, M2 and M3 segments of MCA, A1 and A2 segments of ACA). Proximal occlusions (defined as occlusion of ICA or M1 segment of MCA) were found at 105 patients (71.4%). These patients had more severe strokes (median NIHSS score 15 vs. 8; *P* < 0.001) compared to those with more distal occlusions (M2 and M3 segment of MCA, A1 and A2 segments of ACA), but no significant differences were recorded in leukocyte counts (Table S3 in the Supplementary material). Data on collateral status was available for 145 patients (98.6%). Good collateral circulation was found in 86 patients (59.3%). Patients with poor collateral circulation had higher NIHSS median scores on admission than those with good collaterals (16 vs. 11; P < 0.001), but no significant differences in leukocyte counts were found between the two groups (Table S4 in the Supplementary material).

Twenty patients (12.0%) had LVO in the posterior circulation (VA, BA, P1 and P2 segments of PCA). These patients tended to be younger and had lower median NIHSS scores than patients with LVO in the anterior circulation. Median admission total WBC and neutrophil counts were significantly higher in patients with posterior LVO (*p* = 0.005 and 0.010 respectively). Lymphocyte and monocyte counts were slightly higher in posterior LVO patients; however, differences did not reach the significance level (Table S5 in the Supplementary material).

A total of 100 patients (23.9%) have suffered early post-stroke infections, the majority of which was pneumonia (37%) and urinary tract infections (51%). In the group of non LVO patients on admission neutrophil counts were higher and lymphocyte counts were lower in those with early PSI (*p* = 0.003 and 0.037). However, no differences were found in leukocyte counts according to the development of PSI among patients with LVO (Table S6 in the Supplementary material). No significant differences were recorded in leukocyte counts between the groups of patients with and without hypertension or diabetes. In contrast, lymphocyte, eosinophil and basophil counts were slightly higher in patients with hyperlipidaemia (Table S7 of the Supplementary material).

## Discussion

The main finding of our study is that leukocyte counts (especially total WBC and neutrophil) are associated with the presence of LVO in the acute phase of ischemic stroke. Higher total WBC and neutrophil counts could be detected in LVO patients compared to those without LVO, already in the first hours after stroke onset. This highlights the rapid response of systematic inflammatory mechanisms after ischemic brain injury, the extent of which may differ among leukocyte subtypes according to the presence of LVO.

Proinflammatory factors and pathways are activated within minutes after ischemic onset [12]. Neutrophils are the first leukocyte subtype to be upregulated and subsequently infiltrate the ischemic brain tissue [13]. A previous study has reported that neutrophilia is associated with the volume of ischemic tissue in AIS [5]. The presence of LVO can cause blood supply disturbances in large vascular territories and places substantial cerebral areas under ischemic risk, thereby probably increase the magnitude of proinflammatory response. This may explain why higher total WBC counts (mainly due to the increase in neutrophil counts) can be detected in LVO patients compared to those without LVO in AIS.

Our results are consistent with previous studies highlighting the longitudinal changes in leukocyte activation: elevation of neutrophil and decrease in lymphocyte counts over time [14, 15]. It should be noted that lymphocytes are recruited in the later stages of ischemic brain injury [16]. In our study no differences were found in baseline lymphocyte counts between LVO and non LVO patients, which may be because lymphocytes have not yet been extensively activated at this early stage of AIS. This may also be the reason why NLR, which is well established in stroke prognosis prediction [3, 14, 15], hardly differed between the two groups.

Independent associations between increasing counts of neutrophils, lymphocytes and basophils and higher odds of LVO may represent a broad, bi-directional crosstalk between the ischemic brain and the peripheral immune system, which likely affects almost all participants of the immune response quite early after stroke onset. Interestingly in addition to the strong association between neutrophil counts and LVO, association was also found for basophil counts. Basophil leukocytes have unique role in allergic reactions, parasite infections and autoimmune diseases, however, little data are available on their role in acute stroke. Several years ago, a study has raised the role of basophils in stroke, while another study has confirmed the role of mast cells in regulating the blood-brain barrier following cerebral ischemia [17, 18]. However, it should be noted that automated analysis of leukocyte subtypes with very low number of cells (eosinophil and basophil counts) might be slightly inaccurate. In addition, routine hemogram results (which we also used in this study), despite low concentrations, usually only present two decimal places in the numerical values of absolute basophil counts, hence statistical analysis might be somewhat biased.

Raising the suspicion of LVO in AIS early on is crucial to ensure appropriate imaging methods and early transportation of patients to an EVT capable CSC. Hence reliable blood-based biomarkers would be valuable to detect patients with LVO early on. Our results demonstrated that the ability of leukocyte counts to discriminate the presence of LVO are limited on their own. This may be because changes in peripheral leukocyte counts are not specific for brain damage and can be influenced by many other confounding factors.

Interestingly leukocytes did not associate with the size of the occluded vessel and with the status of collateral circulation in the anterior vascular territory. These findings are partly consistent with the result of a previous study by *Semerano* et al., reporting no significant differences in admission leukocyte counts according to the status of collateral circulation [14]. The interplay between the size of occluded vascular territory and the quality of collateral circulation supplemented by other metabolic and genetic factors are highly related to the size of the core and penumbra within ischemic brain lesions [19, 20]. A study by *Buck* et al. suggests that early changes in peripheral counts are related to the size of bioenergetically compromised brain tissue [5]. Based on our results the magnitude of early peripheral inflammatory response after LVO may not related to the collateral circulation or the size of occluded artery separately. However, the interaction between these factors may affect the size of ischemic core and penumbra, and thus probably the extent of neuroinflammation as well.

A previous study has reported no differences in leukocyte counts between anterior circulation (AC) and posterior circulation (PC) strokes and revealed that NLR values are only correlating with infarct volumes in the AC territory, but not in the PC. However, this study also assessed AIS patients without LVO [21]. The etiology of LVO in the PC and the composition of such thrombi (including the proportion of leukocytes) are different from those of the anterior circulation LVO [22, 23]. It should also be noted that the distribution of neuronal and non-neuronal cells is different in the various areas of the human brain [24, 25], including the proportion of microglia and astrocytes, which may also influence the extent of neuroinflammation. In our study higher median neutrophil and slightly higher lymphocyte and monocyte counts in the posterior LVO group may be related to these conditions. Further studies are needed to assess the relationship between the location of ischemia and the extent of neuroinflammation.

The rapidly evolving, new options in the treatment of AIS due to LVO facilitate the need for better understanding the nature of this type of stroke. Reliable blood based LVO biomarkers would be valuable to detect patients with high likelihood of LVO early on. Such a biomarker could be useful for emergency medical services and emergency department personnel to organize optimal patient pathways and to allocate necessary diagnostic and therapeutic resources as soon as possible. Based on our results leukocyte counts are not sufficiently suitable for this purpose, due to low sensitivity and specificity. However, these findings may warrant further investigation to explore the relationship between LVO and neuroinflammation in details. The scope of further studies could be the interplay between LVO and well-established inflammatory markers such as acute phase proteins, cytokines, cell adhesion molecules, matrix metalloproteinases, damage-associated molecular patterns, markers of oxidative stress, markers of the complement pathway and annexins [1, 26–29]. Inflammatory markers may also be good candidates to find suitable blood-based biomarkers for early LVO detection [30]. Further, larger scale studies are also needed to examine alterations in neuroinflammation according to the location and the volume of cerebral infarction and ischemic penumbra. A recent study has found that NLR values can be useful biomarkers to predict the occurrence of PSI in AIS patients [31]. Although our result only showed differences in NLR values among non LVO patients and no differences were observed in the group of LVO patients. This highlights that the presence of LVO may affect the prognostic ability of NLR to predict PSI, further investigations may be required to clarify this.

As previously discussed, the changes in peripheral leukocyte counts may be epiphenomenal to brain damage. However, previous studies have revealed that higher leukocyte counts in healthy patients are also associated with the increased risk of ischemic stroke events [2, 32]. Further investigation may clarify how peripheral leukocyte counts are related to the risk of suffering an LVO is AIS, or how it may affect the composition of the thrombi.

The main strength of our study is the thorough investigation of multiple leukocyte subtypes in a reasonable number of patients from two university centres. However, our study also has some limitations. The observational, cross-sectional design did not allow to assess cause-effect relationship. No assessment of ischemic lesion volume or of the size of ischemic core and penumbra was made on admission. Although we attempted to exclude patients whose leukocyte counts may be affected by other conditions, we cannot be sure that all such patients have been excluded. There is a chance of other, unknown confounding factors that were not considered in this study. No CTA was performed in almost 8% of screened cases (mainly due to minor symptoms or contraindications), which might lead to selection bias. The small number of patients with posterior LVO resulted a probably underpowered subanalysis. Finally, it is important to emphasize that NIHSS may not appropriately assess the spectrum and severity of PC related neurologic deficits. Therefore, NIHSS scores are usually lower in patients with PC territory strokes than patients with stroke in anterior circulation [33, 34].

## Conclusion

Our study demonstrates that higher on admission total WBC and neutrophil counts are strongly associated with the presence of LVO and has moderate ability to discriminate an LVO in AIS. Further studies are needed to ensure these findings in larger cohorts and to explore the detailed mechanisms of changes in inflammatory pathways after AIS according to the presence of LVO.

## Supplementary Information

**Additional file 1: Table S1.** Univariable associations between baseline characteristics and the presence of LVO in acute ischemic stroke. **Figure S1.** Receiver operating characteristic curves demonstrating the ability of total and differential leukocyte counts to discriminate the presence of LVO in AIS. Area under the curve (AUC) values and 95% confidence intervals are presented. **Table S2.** Capability of leukocyte counts to detect large vessel occlusion in acute ischemic stroke. **Table S3.** Baseline characteristics of patients according to the site of occlusion in the anterior circulation. **Table S4.** Baseline characteristics of patients according to collateral status in the anterior circulation. **Table S5.** Demography and clinical characteristics of LVO patients according to the location of LVO*.* **Table S6.** Differences in leukocyte counts according to the development of early post-stroke infections (PSI). **Table S7.** Differences in leukocyte counts according to the presence of cardiovascular risk factors.

## Data Availability

The datasets used and/or analysed during the current study are available from the corresponding author on reasonable request.

## Abbreviations

AIS: Acute ischemic stroke
LVO: Large vessel occlusion
EVT: Endovascular thrombectomy
CSC: Comprehensive stroke centre
NIHSS: National Institutes of Health Stroke Scale
CTA: Computed tomography angiography
ICA: Internal carotid artery
MCA: Middle cerebral artery
ACA: Anterior cerebral artery
VA: Vertebral artery
BA: Basilar artery
PCA: Posterior cerebral artery
mCTA: multiphase computed tomography angiography
PSI: Post-stroke infection
TIA: Transient ischemic attack
SD: Standard deviation
IQR: Interquartile range
WBC: White blood cell
NLR: Neutrophil-to-lymphocyte ratio
AUC: Area under the curve
OR: Odds ratio
CI: Confidence interval
AC: Anterior circulation
PC: Posterior circulation
