# Supplementary material for: Relationship between leukocyte counts and large vessel occlusion in acute ischemic stroke
Source: BMC Neurol. 2020 Dec 4;20:440. doi: 10.1186/s12883-020-02017-3 (PMC7716438; doi:10.1186/s12883-020-02017-3)
Supplement: Supplementary file 1 — Additional file 1: Table S1. Univariable associations between baseline characteristics and the presence of LVO in acute ischemic stroke. Figure S1. Receiver operating characteristic curves demonstrating the ability of total and differential leukocyte counts to discriminate the presence of LVO in AIS. Area under the curve (AUC) values and 95% confidence intervals are presented. Table S2. Capability of leukocyte counts to detect large vessel occlusion in acute ischemic stroke. Table S3. Baseline characteristics of patients according to the site of occlusion in the anterior circulation. Table S4. Baseline characteristics of patients according to collateral status in the anterior circulation. Table S5. Demography and clinical characteristics of LVO patients according to the location of LVO. Table S6. Differences in leukocyte counts according to the development of early post-stroke infections (PSI). Table S7. Differences in leukocyte counts according to the presence of cardiovascular risk factors. [file 12883_2020_2017_MOESM1_ESM.docx]

**Relationship between leukocyte counts and large vessel occlusion in acute ischemic stroke**

Tarkanyi G, Karadi ZN, Szabo Z, Szegedi I, Csiba L, Szapary L.

**Supplementary material**

**Table S1.** Univariable associations between baseline characteristics and the presence of LVO in acute ischemic stroke

|  | Crude odds ratio  (95% CI) | P value |
| --- | --- | --- |
| **Demographic characteristics** |  |  |
| Age, 1-year increase | 1.011 (0.994 to 1.027) | 0.200 |
| Gender, female gender | 1.738 (1.170 to 2.581) | 0.006* |
| **Elapsed times** |  |  |
| Onset-to-sample time, 1 min increase | 0.998 (0.995 to 1.002) | 0.396 |
| Sample-to-CTA time, 1 min increase | 0.998 (0.988 to 1.008) | 0.715 |
| **Parameters on admission** |  |  |
| NIHSS score on admission, 1-point increase | 1.230 (1.173 to 1.289) | <0.001* |
| On admission SBP, 1 mmHg increase | 0.990 (0.983 to 0.997) | 0.005* |
| On admission DBP, 1 mmHg increase | 0.983 (0.970 to 0.996) | 0.010* |
| Body temperature, 1^0^C increase | 0.437 (0.190 to 1.006) | 0.052­* |
| Blood glucose, 1 mmol/L increase | 1.010 (0.947 to 1.077) | 0.758 |
| INR, 1-point increase | 10.947 (2.383 to 50.287) | 0.002* |
| **Vascular risk factors** |  |  |
| Smoking | 1.401 (0.896 to 2.192) | 0.139 |
| Hypertension | 1.267 (0.770 to 2.085) | 0.353 |
| Diabetes mellitus | 0.626 (0.392 to 1.000) | 0.050* |
| Hyperlipidaemia | 0.887 (0.588 to 1.338) | 0.569 |
| Atrial fibrillation | 2.357 (1.469 to 3.781) | <0.001* |
| Coronary artery disease | 1.258 (0.790 to 2.004) | 0.333 |
| Chronic heart failure | 2.137 (1.118 to 4.084) | 0.022* |
| Previous stroke/TIA | 0.634 (0.384 to 1.048) | 0.075* |
| Malignancy | 1.915 (1.037 to 3.537) | 0.038* |
| **Therapy at stroke onset** |  |  |
| Antiplatelet | 1.201 (0.792 to 1.819) | 0.388 |
| Anticoagulant | 2.003 (1.101 to 3.643) | 0.023* |
| Lipid lowering | 1.330 (0.836 to 2.115) | 0.229 |
| Antihypertensive | 1.345 (0.863 to 2.098) | 0.191 |
| Antidiabetic | 0.620 (0.368 to 1.043) | 0.071* |

Abbreviation: LVO, large vessel occlusion; CI, Confidence Interval, NIHSS, National Institutes of Health Stroke Scale; SBP, systolic blood pressure; DBP, diastolic blood pressure; INR, International Normalized Ratio; TIA, transient ischemic attack.

* Variables included in the multivariable analysis.

***
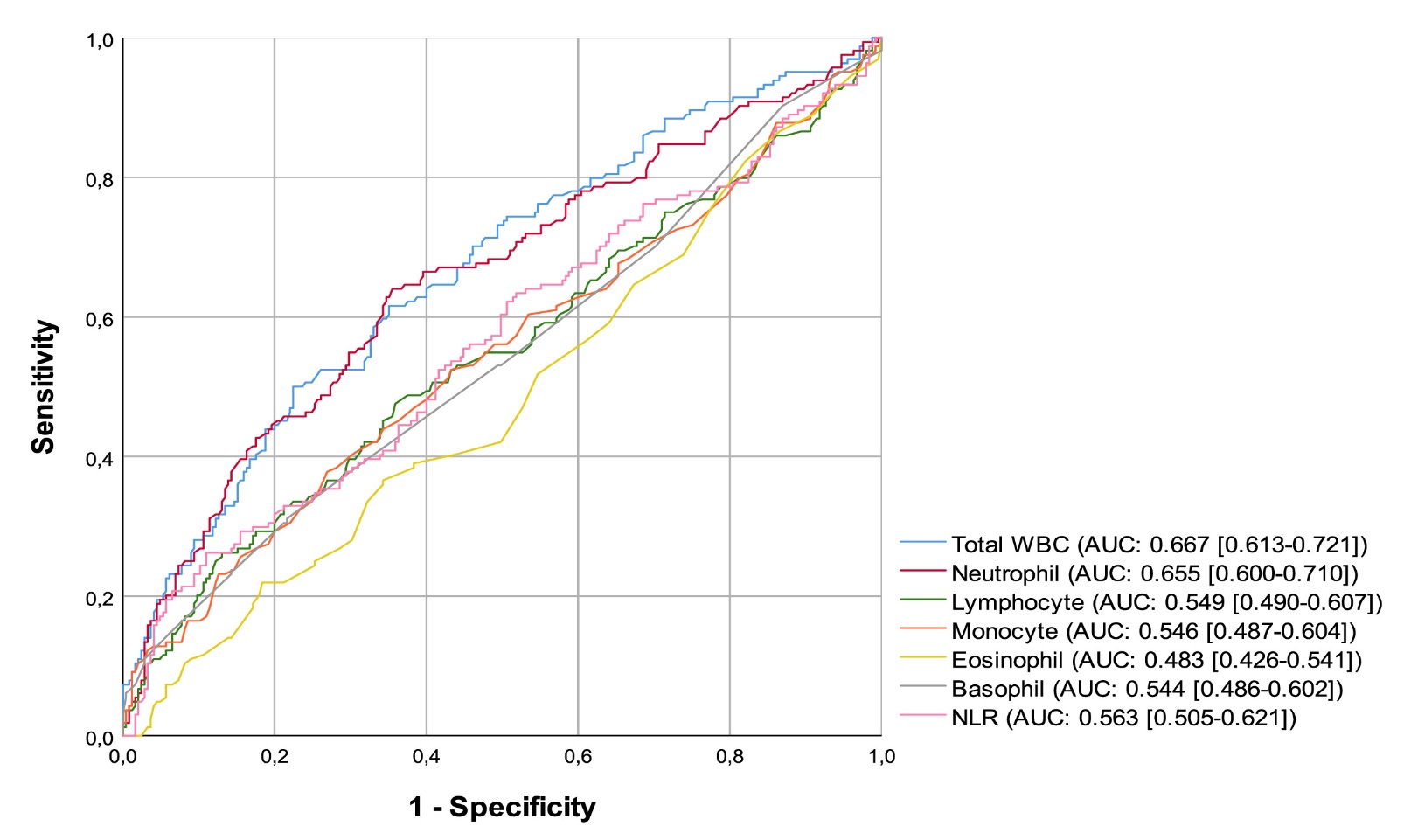
*Figure S1.** Receiver operating characteristic curves demonstrating the ability of total and differential leukocyte counts to discriminate the presence of LVO in AIS. Area under the curve (AUC) values and 95% confidence intervals are presented.

**Table S2.** Capability of leukocyte counts to detect large vessel occlusion in acute ischemic stroke

|  | AUC  (95% CI) | P value | Optimal  cut-off value | Sensitivity | Specificity | PPV | NPV |
| --- | --- | --- | --- | --- | --- | --- | --- |
| Total WBC | 0.667  (0.613 - 0.721) | <0.001 | ≥8.45 G/L | 61.6% | 64.9% | 53,8% | 71,8% |
| Neutrophil | 0.655  (0.600 - 0.710) | <0.001 | ≥5.34 G/L | 64.0% | 64.5% | 54.4% | 73.0% |
| Lymphocyte | 0.549  (0.490-0.607) | 0.093* | - | - | - | - | - |
| Monocyte | 0.546  (0.487-0.604) | 0.116* | - | - | - | - | - |
| Eosinophil | 0.486  (0.426-0.541) | 0.566* | - | - | - | - | - |
| Basophil | 0.544  (0.486-0.602) | 0.133* | - | - | - | - | - |
| NLR | 0.561  (0.504-0.619) | 0.034 | ≥2.43 | 62.9% | 48.0% | 44,5% | 66,1% |

Abbreviation: AUC, area under the curve; CI, confidence interval; WBC, white blood cell; L, litre, PPV, positive predictive value; NPV, negative predictive value.

* Optimal cut-off values and relating sensitivity, specificity, PPV and NPV values were not calculated in variables without significant discrimination ability.

**Table S3.** Baseline characteristics of patients according to the site of occlusion in the anterior circulation

|  | Proximal occlusion  (N=105) | Distal occlusion  (N=42) | P value |
| --- | --- | --- | --- |
| **Demographic characteristics** |  |  |  |
| Age, years, median (IQR) | 69 (62-78) | 70 (64-81) | 0.346 |
| Gender, female, % (n) | 52.3 (55) | 54.8 (23) | 0.794 |
| **Elapsed times** |  |  |  |
| Onset-to-sample time, min, median (IQR) | 75 (52-112) | 91 (70-143) | 0.032 |
| Sample-to-CTA time, min, median (IQR) | 14 (6-22) | 16 (5-28) | 0.985 |
| **Parameters on admission** |  |  |  |
| NIHSS score on admission, median (IQR) | 15 (11-19) | 8 (6-11) | **<0.001** |
| On admission SBP, mmHg, median (IQR) | 159 (136-180) | 154 (140-169) | 0.514 |
| On admission DBP, mmHg, median (IQR) | 84 (76-96) | 89 (80-96) | 0.489 |
| Body temperature, ^o^C, median (IQR) | 36.4 (36.1-36.5) | 36.4 (36.0-36.7) | 0.680 |
| Blood glucose, mmol/L, median (IQR) | 6.77 (5.84-7.92) | 6.57 (5.80-7.94) | 0.874 |
| INR, ratio, median (IQR) | 1.02 (0.95-1.09) | 1.02 (0.99-1.12) | 0.259 |
| **Vascular risk factors** |  |  |  |
| Smoking, % (n), 33 missing | 36.6 (30) | 35.3 (12) | 0.895 |
| Hypertension, % (n), 4 missing | 78.2 (79) | 85.7 (36) | 0.304 |
| Diabetes mellitus, % (n), 8 missing | 22.2 (22) | 17.5 (7) | 0.535 |
| Hyperlipidaemia, % (n), 17 missing | 52.2 (48) | 47.4 (18) | 0.618 |
| Atrial fibrillation, % (n), 9 missing | 31.6 (31) | 45.0 (2) | 0.170* |
| Coronary artery disease, % (n), 10 missing | 28.9 (28) | 30.0 (12) | 0.894 |
| Chronic heart failure, % (n), 7 missing | 15.2 (15) | 17.1 (7) | 0.776 |
| Previous stroke/TIA, % (n), 8 missing | 18.4 (18) | 14.6 (6) | 0.595 |
| Malignancy, % (n), 13 missing | 18.8 (18) | 15.8 (6) | 0.687 |
| **Therapy at stroke onset** |  |  |  |
| Antiplatelet, % (n), 9 missing | 46.9 (45) | 30.0 (12) | 0.069 |
| Anticoagulant, % (n), 12 missing | 14.4 (14) | 31.6 (12) | 0.023 |
| Lipid lowering, % (n), 10 missing | 29.2 (28) | 24.4 (10) | 0.567 |
| Antihypertensive, % (n), 10 missing | 71.9 (69) | 75.6 (31) | 0.652 |
| Antidiabetic, % (n), 11 missing | 17.7 (17) | 15.0 (6) | 0.701 |
| **Leukocyte counts (1x10^9^/L)** |  |  |  |
| Total WBC, median (IQR) | 8.75 (7.13-11.00) | 9.13 (7.22-10.42) | 0.585 |
| Neutrophil, median (IQR) | 5.93 (4.29-7.57) | 5.79 (3.87-7.66) | 0.646 |
| Lymphocyte, median (IQR) | 1.97 (1.38-2.67) | 1.75 (1.33-2.84) | 0.528 |
| Monocyte, median (IQR) | 0.67 (0.49-0.83) | 0.62 (0.50-0.79) | 0.759 |
| Eosinophil, median (IQR) | 0.12 (0.07-0.21) | 0.12 (0.07-0.20) | 0.666 |
| Basophil, median (IQR) | 0.05 (0.03-0.07) | 0.05 (0.04-0.07) | 0.642 |
| **NLR, median (IQR)** | 2.79 (1.99-4.73) | 2.79 (1.95-6.04) | 0.959 |

Abbreviation: LVO, large vessel occlusion; NIHSS, National Institutes of Health Stroke Scale; SBP, systolic blood pressure; DBP, diastolic blood pressure; IQR, interquartile range; INR, International Normalized Ratio; TIA, transient ischemic attack; NLR, neutrophil-to-lymphocyte ratio.

Proximal occlusion: ICA and MCA M1; distal occlusion: MCA M2, M3 and ACA A1 and A2

* Fischer exact test

**Table S4.** Baseline characteristics of patients according to collateral status in the anterior circulation

|  | Good collaterals  (N=86) | Poor collaterals (N=56) | P value |
| --- | --- | --- | --- |
| **Demographic characteristics** |  |  |  |
| Age, years, median (IQR) | 69 (62-79) | 71 (62-81) | 0.448 |
| Gender, female, % (n) | 52.3 (55) | 54.8 (23) | 0.794 |
| **Elapsed times** |  |  |  |
| Onset-to-sample time, min, median (IQR) | 79 (50-128) | 83 (60-115) | 0.594 |
| Sample-to-CTA time, min, median (IQR) | 13 (5-22) | 17 (6-26) | 0.256 |
| **Parameters on admission** |  |  |  |
| NIHSS score on admission, median (IQR) | 11 (6-16) | 16 (12-19) | **<0.001** |
| On admission SBP, mmHg, median (IQR) | 158 (139-170) | 155 (140-179) | 0.685 |
| On admission DBP, mmHg, median (IQR) | 82 (78-92) | 90 (80-99) | 0.337 |
| Body temperature, ^o^C, median (IQR) | 36.4 (36.0-36.5) | 36.4 (36.1-36.5) | 0.876 |
| Blood glucose, mmol/L, median (IQR) | 6.63 (5.90-7.70) | 6.69 (5.75-8.60) | 0.943 |
| INR, ratio, median (IQR) | 1.00 (0.95-1.07) | 1.03 (0.97-1.12) | 0.141 |
| **Vascular risk factors** |  |  |  |
| Smoking, % (n), 30 missing | 36.6 (26) | 34.1 (15) | 0.783 |
| Hypertension, % (n), 4 missing | 82.9 (68) | 76.3 (45) | 0.328 |
| Diabetes mellitus, % (n), 8 missing | 23.1 (18) | 18.6 (11) | 0.529 |
| Hyperlipidaemia, % (n), 17 missing | 54.7 (41) | 47.2 (25) | 0.403 |
| Atrial fibrillation, % (n), 11 missing | 35.4 (28) | 36.8 (21) | 0.867 |
| Coronary artery disease, % (n), 10 missing | 28.2 (22) | 31.6 (12) | 0.672 |
| Chronic heart failure, % (n), 7 missing | 12.5 (10) | 20.7 (12) | 0.195 |
| Previous stroke/TIA, % (n), 8 missing | 17.5 (14) | 17.5 (10) | 0.995 |
| Malignancy, % (n), 13 missing | 16.9 (13) | 20.0 (11) | 0.647 |
| **Therapy at stroke onset** |  |  |  |
| Antiplatelet, % (n), 10 missing | 41.0 (32) | 43.9 (25) | 0.742 |
| Anticoagulant, % (n), 11 missing | 18.2 (14) | 21.2 (12) | 0.678 |
| Lipid lowering, % (n), 10 missing | 30.8 (24) | 24.6 (14) | 0.428 |
| Antihypertensive, % (n), 10 missing | 79.5 (58) | 71.9 (41) | 0.753 |
| Antidiabetic, % (n), 10 missing | 20.5 (16) | 12.3 (7) | 0.209 |
| **Leukocyte counts (1x10^9^/L)** |  |  |  |
| Total WBC, median (IQR) | 9.23 (7.84-10.97) | 8.55 (6.50-10.42) | 0.105 |
| Neutrophil, median (IQR) | 6.04 (4.47-7.57) | 5.64 (3.87-7.54) | 0.198 |
| Lymphocyte, median (IQR) | 1.96 (1.36-2.86) | 1.97 (1.25-2.40) | 0.226 |
| Monocyte, median (IQR) | 0.67 (0.50-0.87) | 0.61 (0.48-0.78) | 0.203 |
| Eosinophil, median (IQR) | 0.13 (0.07-0.24) | 0.12 (0.07-0.18) | 0.998 |
| Basophil, median (IQR) | 0.05 (0.03-0.08) | 0.04 (0.03-0.06) | 0.360 |
| **NLR, median (IQR)** | 2.69 (1.97-5.08) | 2.83 (1.85-5.38) | 0.822 |

Abbreviation: LVO, large vessel occlusion; NIHSS, National Institutes of Health Stroke Scale; SBP, systolic blood pressure; DBP, diastolic blood pressure; IQR, interquartile range; INR, International Normalized Ratio; TIA, transient ischemic attack; NLR, neutrophil-to-lymphocyte ratio.

**Table S5.** Demography and clinical characteristics of LVO patients according to the location of LVO

|  | Anterior LVO  (N=147) | Posterior LVO  (N=20) | P value |
| --- | --- | --- | --- |
| **Demographic characteristics** |  |  |  |
| Age, years, median (IQR) | 69 (62-79) | 62 (60-69) | **0.016** |
| Gender, female, % (n) | 53.1 (78) | 45.0 (9) | 0.498 |
| **Elapsed times** |  |  |  |
| Onset-to-sample time, min, median (IQR) | 82 (54-119) | 94 (61-160) | 0.244 |
| Sample-to-CTA time, min, median (IQR) | 14 (6-24) | 22 (10-31) | 0.060 |
| **Parameters on admission** |  |  |  |
| NIHSS score on admission, median (IQR) | 13 (8-18) | 6 (4-12) | **0.004** |
| On admission SBP, mmHg, median (IQR) | 158 (140-174) | 165 (143-190) | 0.213 |
| On admission DBP, mmHg, median (IQR) | 85 (80-96) | 80 (74-99) | 0.767 |
| Body temperature, ^o^C, median (IQR) | 36.4 (36.1-36.5) | 36.5 (36.2-36.5) | 0.871 |
| Blood glucose, mmol/L, median (IQR) | 6.70 (5.81-7.94) | 7.63 (6.49-9.97) | **0.014** |
| INR, ratio, median (IQR) | 1.02 (0.95-1.09) | 0.99 (0.96-1.03) | 0.087 |
| **Vascular risk factors** |  |  |  |
| Smoking, % (n), 34 missing | 36.2 (42) | 58.8 (10) | 0.104* |
| Hypertension, % (n), 4 missing | 80.4 (115) | 90.0 (18) | 0.374* |
| Diabetes mellitus, % (n), 8 missing | 20.9 (29) | 25.0 (5) | 0.771* |
| Hyperlipidaemia, % (n), 17 missing | 50.8 (66) | 50.0 (10) | 0.949 * |
| Atrial fibrillation, % (n), 9 missing | 35.6 (49) | 15.0 (3) | **0.078*** |
| Coronary artery disease, % (n), 11 missing | 29.2 (40) | 16.7 (3) | 0.402* |
| Chronic heart failure, % (n), 7 missing | 15.7 (22) | 10.0 (2) | 0.741* |
| Previous stroke/TIA, % (n), 8 missing | 17.3 (24) | 25.0 (4) | 0.756* |
| Malignancy, % (n), 15 missing | 17.9 (24) | 5.6 (1) | 0.310* |
| **Therapy at stroke onset** |  |  |  |
| Antiplatelet, % (n), 13 missing | 41.9 (57) | 27.8 (5) | 0.312* |
| Anticoagulant, % (n), 14 missing | 19.3 (26) | 5.6 (1) | 0.200* |
| Lipid lowering, % (n), 12 missing | 27.7 (38) | 27.8 (5) | 0.997* |
| Antihypertensive, % (n), 12 missing | 73.0 (100) | 72.2 (13) | 0.945* |
| Antidiabetic, % (n), 14 missing | 16.9 (23) | 11.8 (2) | 0.741* |
| **Leukocyte counts (1x10^9^/L)** |  |  |  |
| Total WBC, median (IQR) | 8.77 (7.13-10.97) | 10.46 (9.30-12.41) | **0.005** |
| Neutrophil, median (IQR) | 5.89 (4.20-7.60) | 7.06 (5.98-9.99) | **0.010** |
| Lymphocyte, median (IQR) | 1.94 (1.34-2.70) | 2.22 (1.60-3.32) | 0.224 |
| Monocyte, median (IQR) | 0.64 (0.49-0.83) | 0.76 (0.54-0.93) | 0.114 |
| Eosinophil, median (IQR) | 0.12 (0.07-0.20) | 0.09 (0.05-0.21) | 0.226 |
| Basophil, median (IQR) | 0.05 (0.03-0.07) | 0.05 (0.04-0.08) | 0.682 |
| **NLR, median (IQR)** | 2.79 (1.97-5.28) | 3.13 (2.29-5.44) | 0.344 |

Abbreviation: LVO, large vessel occlusion; NIHSS, National Institutes of Health Stroke Scale; SBP, systolic blood pressure; DBP, diastolic blood pressure; IQR, interquartile range; INR, International Normalized Ratio; TIA, transient ischemic attack; NLR, neutrophil-to-lymphocyte ratio.

* Fischer exact test.

**Table S6.** Differences in leukocyte counts according to the development of early post-stroke infections (PSI)

| Leukocyte counts (1x10^9^/L) | No early PSI developed | Early PSI developed | P value |
| --- | --- | --- | --- |
| **Part A** Overall (n=419) | n=319 (76.1%) | n=100 (23.9%) |  |
| Total WBC, median (IQR) | 8.15 (6.58-9.91) | 8.29 (6.69-10.09) | 0.625 |
| Neutrophil, median (IQR) | 5.03 (3.86-6.87) | 5.78 (4.09-6.89) | 0.140 |
| Lymphocyte, median (IQR) | 1.93 (1.34-2.62) | 1.77 (1.19-2.32) | **0.020** |
| Monocyte, median (IQR) | 0.63 (0.49-0.79) | 0.64 (0.53-0.82) | 0.494 |
| Eosinophil, median (IQR) | 0.13 (0.07-0.21) | 0.13 (0.07-0.20) | 0.955 |
| Basophil, median (IQR) | 0.05 (0.03-0.06) | 0.04 (0.03-0.07) | 0.957 |
| NLR, median (IQR) | 2.56 (1.76-4.00) | 2.98 (2.07-4.97) | **0.013** |
| **Part B** LVO absent (n=252) | n=196 (77.8%) | n=56 (22.2%) |  |
| Total WBC, median (IQR) | 7.34 (6.20-9.02) | 8.24 (6.61-9.65) | 0.050 |
| Neutrophil, median (IQR) | 4.52 (3.57-5.78) | 5.73 (4.20-6.61) | **0.003** |
| Lymphocyte, median (IQR) | 1.90 (1.34-2.45) | 1.65 (1.15-2.11) | **0.037** |
| Monocyte, median (IQR) | 0.61 (0.49-0.76) | 0.62 (0.53-0.83) | 0.298 |
| Eosinophil, median (IQR) | 0.13 (0.07-0.20) | 0.14 (0.06-0.22) | 0.456 |
| Basophil, median (IQR) | 0.04 (0.03-0.06) | 0.04 (0.03-0.06) | 0.560 |
| NLR, median (IQR) | 2.37 (1.75-3.51) | 3.04 (2.17-5.09) | **0.002** |
| **Part C** LVO present (n=167) | n=123 (73.7%) | n=44 (26.3%) |  |
| Total WBC, median (IQR) | 9.42 (7.70-11.53) | 8.50 (6.92-10.16) | 0.084 |
| Neutrophil, median (IQR) | 6.21 (4.47-8.12) | 5.79 (3.98-7.63) | 0.246 |
| Lymphocyte, median (IQR) | 2.15 (1.36-2.91) | 1.81 (1.35-2.48) | 0.165 |
| Monocyte, median (IQR) | 0.67 (0.49-0.84) | 0.64 (0.53-0.81) | 0.803 |
| Eosinophil, median (IQR) | 0.12 (0.06-0.24) | 0.12 (0.08-0.18) | 0.503 |
| Basophil, median (IQR) | 0.05 (0.03-0.07) | 0.04 (0.03-0.07) | 0.426 |
| NLR, median (IQR) | 2.83 (1.95-5.42) | 2.88 (2.05-4.79) | 0.874 |

Abbreviation: PSI, post-stroke infection; WBC, white blood cell; IQR, interquartile range; NLR, neutrophil-to-lymphocyte ratio; LVO, large vessel occlusion.

**Table S7.** Differences in leukocyte counts according to the presence of cardiovascular risk factors

| Leukocyte counts (1x10^9^/L) | Risk factor absent | Risk factor present | P value |
| --- | --- | --- | --- |
| **Hypertension** (n=406) | n=84 (20.7%) | n=322 (79.3%) |  |
| Total WBC, median (IQR) | 8.25 (6.22-9.68) | 8.19 (6.67-9.94) | 0.677 |
| Neutrophil, median (IQR) | 5.04 (3.73-7.00) | 5.27 (3.96-6.89) | 0.651 |
| Lymphocyte, median (IQR) | 1.81 (1.38-2.46) | 1.89 (1.31-2.55) | 0.887 |
| Monocyte, median (IQR) | 0.60 (0.51-0.80) | 0.64 (0.49-0.79) | 0.910 |
| Eosinophil, median (IQR) | 0.12 (0.06-0.20) | 0.13 (0.07-0.20) | 0.705 |
| Basophil, median (IQR) | 0.05 (0.03-0.07) | 0.04 (0.03-0.06) | 0.208 |
| NLR, median (IQR) | 2.76 (1.84-3.84) | 2.69 (1.86-4.15) | 0.967 |
| **Diabetes** (n=400) | n=293 (73.3%) | n=107 (26.7%) |  |
| Total WBC, median (IQR) | 8.12 (6.55-9.87) | 8.29 (6.90-10.43) | 0.283 |
| Neutrophil, median (IQR) | 5.04 (3.87-6.87) | 5.23 (4.07-6.93) | 0.512 |
| Lymphocyte, median (IQR) | 1.85 (1.31-2.47) | 1.97 (1.41-2.66) | 0.135 |
| Monocyte, median (IQR) | 0.62 (0.49-0.79) | 0.62 (0.52-0.80) | 0.528 |
| Eosinophil, median (IQR) | 0.11 (0.06-0.20) | 0.14 (0.08-0.21) | 0.193 |
| Basophil, median (IQR) | 0.04 (0.03-0.06) | 0.05 (0.03-0.07) | 0.160 |
| NLR, median (IQR) | 2.73 (1.95-4.15) | 2.63 (1.75-3.89) | 0.409 |
| **Hyperlipidaemia** (n=383) | n=182 (47.5%) | n=201 (52.5%) |  |
| Total WBC, median (IQR) | 8.11 (6.46-10.30) | 8.30 (6.62-9.65) | 0.911 |
| Neutrophil, median (IQR) | 5.33 (3.75-7.10) | 4.94 (3.98-6.53) | 0.395 |
| Lymphocyte, median (IQR) | 1.78 (1.22-2.38) | 1.91 (1.42-2.58) | 0.019 |
| Monocyte, median (IQR) | 0.60 (0.49-0.79) | 0.64 (0.51-0.78) | 0.402 |
| Eosinophil, median (IQR) | 0.11 (0.07-0.18) | 0.14 (0.07-0.22) | 0.030 |
| Basophil, median (IQR) | 0.04 (0.03-0.06) | 0.05 (0.03-0.07) | 0.043 |
| NLR, median (IQR) | 2.78 (1.88-4.55) | 2.56 (1.80-3.58) | 0.058 |

Abbreviation: PSI, post-stroke infection; WBC, white blood cell; IQR, interquartile range; NLR, neutrophil-to-lymphocyte ratio.
